# CD19 CAR-T cell treatment conferred sustained remission in B-ALL patients with minimal residual disease

**DOI:** 10.1007/s00262-021-02941-4

**Published:** 2021-04-25

**Authors:** Wenyi Lu, Yunxiong Wei, Yaqing Cao, Xia Xiao, Qing Li, Hairong Lyu, Yili Jiang, Huan Zhang, Xin Li, Yanyu Jiang, Juanxia Meng, Ting Yuan, Haibo Zhu, Xiaoyuan He, Xin Jin, Rui Sun, Tao Sui, Kaiqi Liu, Mingfeng Zhao

**Affiliations:** 1 Department of Hematology, Tianjin First Central Hospital, No. 24 Fu Kang Road, Tianjin, 300192 People’s Republic of China; 2 Nankai University Affiliated First Central Hospital, No. 24 Fu Kang Road, Tianjin, 300192 People’s Republic of China; 3 School of Medicine, Nankai University, No.94 Weijin Road, Tianjin, 300071 People’s Republic of China; 4 Leukemia Center, Institute of Hematology and Blood Diseases Hospital, Peking Union Medical College and Chinese Academy of Medical Sciences, 288 Nanjing Road, Tianjin, 300060 People’s Republic of China

**Keywords:** B-cell acute lymphoblastic leukemia, Minimal residual disease, CD19-directed chimeric antigen receptor T cells, Cytokine release syndrome, Relapse

## Abstract

The persistence or recurrence of minimal residual disease (MRD) after chemotherapy predicts relapse of B-cell acute lymphoblastic leukemia (B-ALL). CD19-directed chimeric antigen receptor T (CD19 CAR-T) cells have shown promising responses in B-ALL. However, their role in chemotherapy-refractory MRD-positive B-ALL remains unclear. Here we aimed to assess the effectiveness and safety of CD19 CAR-T cells in MRD-positive B-ALL patients. From January 2018, a total of 14 MRD-positive B-ALL patients received one or more infusions of autogenous CD19 CAR-T cells. Among them, 12 patients achieved MRD-negative remission after one cycle of CAR-T infusion. At a median follow-up time of 647 days (range 172–945 days), the 2-year event-free survival rate in MRD-positive patients was 61.2% ± 14.0% and the 2-year overall survival was 78.6 ± 11.0%, which were significantly higher than patients with active disease (blasts ≥ 5% or with extramedullary disease). Moreover, patients with MRD had a lower grade of cytokine release syndrome (CRS) than patients with active disease. However, the peak expansion of CAR-T cells in MRD positive patients showed no statistical difference compared to patients with active disease. Five patients received two or more CAR-T cell infusions and these patients showed a decreased peak expansion of CAR-T cell in subsequent infusions. In conclusion, pre-emptive CD19 CAR-T cell treatment is an effective and safe approach and may confer sustained remission in B-ALL patients with chemotherapy-refractory MRD. The trials were registered at www.chictr.org.cn as ChiCTR-ONN-16009862 (November 14, 2016) and ChiCTR1800015164 (March 11, 2018).

**Supplementary Information:**

The online version contains supplementary material available at 10.1007/s00262-021-02941-4.

## Introduction

Relapse is a frequent cause of treatment failure in B-cell acute lymphoblastic leukemia (B-ALL) patients. Minimal residual disease (MRD) is an essential predictor of hematopoietic relapse. The persistence or recurrence of MRD after chemotherapy indicates resistance to chemotherapeutic drugs and is a poor prognostic indicator for adult B-ALL patients [1, 2]. In standard-risk adult ALL, the median relapse-free survival in patients converting to MRD-positive during the early postconsolidation phase is 16.7 months, which is shorter than that for patients with continuously negative MRD. Allogeneic hematopoietic stem cell transplantation (allo-HSCT) is the most effective method to cure B-ALL. However, transplantation related mortality and complications are still major obstacles. Thus, novel treatment for these patients is needed.

CD19-directed chimeric antigen receptor T (CD19 CAR-T) cell is a new modality for the treatment of B-ALL, B-cell lymphoma and chronic lymphocytic leukemia [3–5]. In relapsed/refractory B-ALL patients, the overall complete remission (CR) rate of CAR-T therapy is 80%, and CAR-T has also been shown to be effective in achieving MRD negativity [6, 7]. Some studies have shown that subjects with low ALL burden have a higher CR rate than those with high ALL burden, which indicates that CAR-T therapy may achieve a better response in MRD-positive patients than refractory/relapsed B-ALL patients [7, 8]. It should be specifically noted that in the study of Park et al., the subgroup with lower tumor burden (blasts representing between 0 and 5% of bone marrow cells) had a higher CR rate and lower risk of CRS than the subgroup with a higher disease burden (≥ 5% bone marrow blasts or extramedullary disease), although these findings did not reach statistical significance. These studies indicate that CAR-T therapy may achieve a better response in MRD-positive patients than refractory/relapsed B-ALL patients [7]. Moreover, a retrospective study showed that donor-derived CD19 CAR-T cells could eliminate B-ALL MRD after HSCT [9]. By accurately identifying patients with persistent MRD who are at highest risk for relapse, we may treat these patients more effectively using CD19 CAR-T cells.

We conduct this study to investigate whether CAR-T cells could eliminate residual leukemia cells and prevent clinical relapse in patients with MRD-positive B-ALL. In this study, the safety and efficacy of autologous CD19 CAR-T cells were assessed in 14 MRD–positive B-ALL patients. We found that CAR-T cells could induce long-lasting molecular remission in B-ALL patients with persistent or recurrent MRD. More importantly, CAR-T cell infusion without subsequent transplantation also led to improved survival. Furthermore, the treatment was well tolerated, as the instances of cytokine release syndrome (CRS) were moderate in these patients. These results suggest that preemptive intervention with CAR-T cells in MRD-positive patients might be a rational option for patients who are unable to receive allo-HSCT or intensive chemotherapy.

## Methods

### Patients and eligibility

From January 2018, a total of 14 B-ALL patients in CR with persistent or recurrent MRD were enrolled in CAR-T cell clinical trials ChiCTR-ONN-16009862 and ChiCTR1800015164. The enrolled patients came from two different clinical trials of CAR-T cells, and the characteristics of the entire cohort are summarized in Table 1. Twenty-nine patients who had active disease (blasts ≥ 5% or with extramedullary disease) from the entire cohort were considered as control group. Patients who were treated in an MRD-positive state were considered as study group in this analysis: 1) if patients were in a consistent MRD-positive state after consolidation chemotherapy, they were categorized into the MRD refractory subgroup; 2) if the patients achieved MRD negativity previously and then converted to MRD positive, they were categorized into the MRD relapse subgroup. Flow cytometry (FCM), real-time quantitative polymerase chain reaction (RQ-PCR) and/or next-generation sequencing (NGS) were used to evaluate MRD. The exclusion criteria were as follows: central nervous system leukemia; pregnant and lactating women; receipt of any investigational drugs within 3 months; prior allogeneic or autologous HSCT; autoimmune disease; and radiotherapy. None of the patients had previously received anti-CD19 therapies before (e.g., blinatumomab).

**Table 1:** Baseline characteristics of the entire cohort of 47 B-ALL patients receiving CAR-T

Age (Median, range)-yr	29 (13–66)
Male sex-no.- (%)	27 (57.4%)
Bone marrow blasts before CAR-T- no. (%)	
Median (range)	30% (0–98.8%)
< 0.01%	4 (8.5%)
0.01–5% (MRD-positive)	14 (29.8%)
≥ 5% or with extramedullary disease	29 (61.7%)
Central nervous system leukemia- no. (%)	6 (12.8%)
Complex chromosome aberration- no. (%)	9 (19.1%)
Ph chromosome-positive- no. (%)	12 (25.5%)

This study was approved by the ethics commission of Tianjin First Center Hospital. All participants provided written informed consent. This analysis reflects all patients with a day cutoff date of August 19, 2020.

### CD19 CAR-T cell infusion

CD19 CAR-T cells were manufactured as previously reported [5]. Briefly, T lymphocytes were enriched from peripheral blood mononuclear cells using CD3 microbeads (Miltenyi Biotec, Germany). The expansion of CAR-T cells was stimulated by adding CD3/CD28 beads and IL-2. T lymphocytes were then transduced with recombinant lentiviral vectors encoding the CD19-28ζ CAR gene and expanded for another 10 days. The percentage of cells with successful CAR transduction in all patients was approximately 50%. Before CAR-T cell infusion, cyclophosphamide was given at 750 mg/m^2^ (day-2) and fludarabine was given at 30 mg/m^2^ for three days. The infused CAR-T cell dose was at least 1 × 10^6^/kg. Patients received repeat CAR-T cell infusion if they had relapsed or they had refractory MRD but did not undergo transplantation. In the latter condition, the interval of CAR-T cell infusion was six months.

### Efficacy assessments

Bone marrow examination was performed on the 14, 28 and 60th days after CAR-T cell infusion. Tests were then repeated every 2 months or more frequently if clinically indicated. MRD was monitored by FCM, RQ-PCR or deep sequencing for the malignant clone with BM aspirate samples. A panel of antibodies that recognize CD10, CD13, CD19, CD20, CD22, CD33, CD34, CD38, CD45, cTdT, and cCD79a was used for MRD detection. The samples were acquired on a Beckman Coulter Cytoflex. FCM positivity was defined as > 0.01% of cells with a leukemia-associated antigen phenotype in bone marrow. PCR positivity was defined as > 0.01% expression of BCR-ABL. TP53, C-KIT, TET2 and IKZF1 mutations in bone marrow were evaluated by NGS analysis. MRD-negative remission was defined as complete hematopoietic remission and negativity on FCM, RQ-PCR and NGS.

### Assessment of adverse events after CAR-T cell infusion

CRS and other adverse events were evaluated in the patients according to a previous study and Common Terminology Criteria for Adverse Events version 4.0 [10, 11]. Furthermore, the concentrations of serum inflammatory markers including C-reactive protein (CRP), IL-2R, IL-6, IL-8, IL-10 and ferritin were assessed by Luminex assay, according to the manufacturer’s protocol.

### Assessment of lymphocyte subsets

The assessment of lymphocyte subsets was performed as described previously [12]. Peripheral blood mononuclear cells were stained using antibody panel for lymphocyte subsets and analyzed by CytExpert software (Beckman Coulter).

### Statistical analysis

The results regarding patient characteristics were obtained using descriptive statistics. The Mann–Whitney U test or Student’s t test was used to compare the absolute number of T cell subsets between different groups. Two-year overall survival (OS) and two-year event-free survival (EFS) were calculated using Kaplan–Meier analysis. OS was defined as the time from CAR-T cell treatment to death. EFS was defined as the time from CAR-T cell treatment to the date of progression or death. Statistical analyses were performed using the SPSS version 19.0 software (Chicago, IL, USA).

## Results

### Demographic and clinical characteristics

From January 2018, a total of 14 B-ALL patients with relapsed/refractory MRD were treated with CD19 CAR-T cells. All patients had detectable MRD after initial therapy and were in CR. The characteristics of the patients and infused cells are described in Table 2. The median dose of infused CAR-T cells was 6.78 × 10^6^ cells/kg, and five patients received more than one infusion. The median follow-up was 647 days (range 172–945 days). Eight patients had persistent MRD and six had MRD relapse. Among them, five patients had Ph-positive B-ALL and were refractory to imatinib or dasatinib before CAR-T cell therapy. They all underwent allo-HSCT immediately after CAR-T cell therapy.

**Table 2:** Characteristics of MRD-positive patients and CAR-T cells

Patient	Age, years	Sex	Maliganancy	Detection of MRD	MRD refractory/Relapse	No. of prior therapies	Last line of therapy	CAR-T cell infused/kg	Frequency of CAR-T infusion	MRD evaluation at 28th day after infusion	Additional therapy after CAR-T	CRS grade	Clinical outcomes
1	46	M	B-ALL Ph+	FCM, BCR/ABL	Relapse	8	VDCLP	5.84×10^6^	1	MR	Son/Haplo-HSCT(5/10)	1	MR
2	13	F	B-ALL	FCM	Refractory	2	CAMVL	2.60×10^6^	2	MR	Consolidation& maintenance therapy	0	MR
3	19	M	B-ALL	FCM	Refractory	2	CAMVL	2.37×10^6^	2	MR	Consolidation& maintenance therapy	1	MR
4	17	F	B-ALL	FCM	Relapse	14	VP	5.57×10^6^	3	MR	No	0	Relapse
5	27	M	B-ALL	FCM	Refractory	3	FLAG	6.98×10^6^	1	MR	Consolidation therapy&Consolidation therapy&Father /Haplo –HSCT (5/10)	1	Relapse
6	40	F	B-ALL Ph+	FCM, BCR/ABL	Refractory	3	FLAG	8.77×10^6^	1	MR	Daughter/Haplo -HSCT (5/10)	1	MR
7	62	M	B-ALL TET2 mutation	FCM, TET2	Relapse	10	VDCP	7.13×10^6^	3	MR	No	1	Relapse
8	41	F	B-ALL	FCM	Relapse	11	VMMP	6.57×10^6^	1	MR	No	1	MR
9	17	M	B-ALL	FCM	Refractory	2	CAMVL	9.22×10^6^	3	MR	Consolidation therapy	1	Relapse (death)
10	42	F	B-ALL Ph+, TP53+ , C-kit+	FCM, BCR/ABL, TP53	Refractory	4	VMCP + Imatinib	9.25×10^6^	1	MR	Sibling HSCT	0	MR
11	29	M	B-ALL IKZF1 +	FCM, IKZF1	Refractory	4	EOACP	10.4×10^6^	1	No response	Father /Haplo -HSCT (5/10)	1	MR
12	45	F	B-ALL Ph+ , IKZF1 +	FCM, BCR/ABL, IKZF1	Refractory	5	VTCD + Dasatinib	8.77×10^6^	1	No response	Sibling HSCT	1	MR
13	38	M	B-ALL	FCM	Relapse	8	VDLD	2.2×10^6^	1	MR	No	1	MR
14	37	F	B-ALL Ph+	FCM, BCR/ABL	Relapse	8	VDCP + Dasatinib	3.1×10^6^	1	MR	Sibling HSCT	0	MR

*F* female, *M* male, *No.* number, *Haplo-HSCT* haploidentical hematopoietic stem cell transplantation, *MR* molecular remission. *VP* vincristine+ dexamethasone, *CAMVL* cyclophosphamide+ cytarabine+ 6-mereaptopurine+ vincristine/vindesine+ L-asparaginase, *FLAG* fludarabine+ cytarabine+ granulocyte colony-stimulating factor, *VDCP* vincristine/vindesine + daunorubicin+ cyclophosphamide + dexamethasone, *VMMP* vincristine/vindesine+ prednison+ 6-mereaptopurine+ methotrexate, *VMCP* vincristine/vindesine+ mitoxantrone+ Ifosfamide+ prednison, *EOACP* VP-16+ vincristine+ cytarabine+ cyclophosphamide+ prednison, *VTCD* vincristine+ VM-26 +cyclophosphamide+ dexamethasone, *VDLD* vincristine/vindesine + daunorubicin+ L-asparaginase+ dexamethasone

### Efficacy

**Fig. 1:**
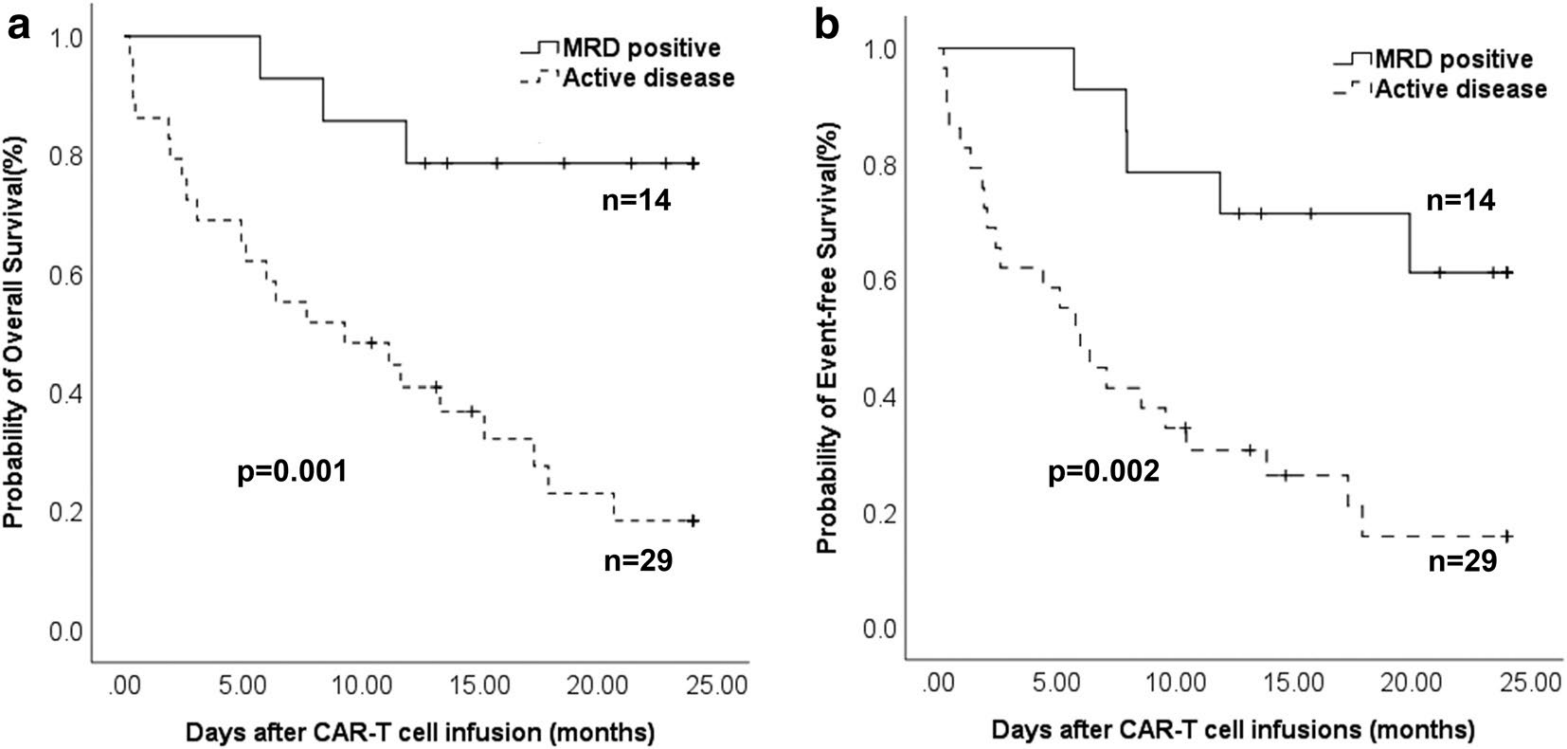
The probability of two-year overall survival and event-free survival in MRD-positive patients and the patients with active disease treated after CAR-T cell therapy. **a** The probability of two-year overall survival after CAR-T cell therapy; **b** The probability of two-year event-free survival after CAR-T cell therapy

One month after the infusion of CAR-T cells, conversion to MRD negativity was observed in 12 patients, leading to a response rate of 85.7% (supplementary Fig. 1). At a median follow-up time of 647 days, 12 patients were in persistent CR, resulting in a 2-year EFS rate of 61.2% ± 14.0%. The probability of 2-year OS was 78.6 ± 11.0%. The 2-year OS and EFS in patients with MRD were significantly higher in comparison to the patients with active disease (Fig. 1).

Eight of nine BCR-ABL negative patients achieved a MRD-negative CR after one cycle of CAR-T cell therapy. Four patients remained in continuous remission, with a duration of response averaging 22.9 months (range: 12.1–28.6 months). Of the four patients who experienced a morphologic relapse, one patient (Patient 5) experienced a CD19-negative relapse and the other three patients (Patients 4, 7, and 9) experienced CD19-positive relapse. Median time to relapse was 418 days (range: 238–740 days). It should be noted that the MRD level of the three relapsed patients (Patients 5, 7, and 9) before CAR-T cell therapy was higher than that of the other patients. Pearson correlation analysis showed that the MRD level was significantly correlated with peak expansion of CAR-T cells (*r* = 0.640, *p* = 0.014) and duration time of response (*r* = − 0.537, *p* = 0.048). Patient 5 developed MRD positivity 123 days after CAR-T cell infusion, and then he received two courses of chemotherapy as consolidation therapy. Unfortunately, he suffered morphologic relapse with CD19-negative blasts at 238 days after CAR-T cell infusion. He then received one cycle of FLAG plus mitoxantrone with which he achieved MRD-positive remission and proceeded to allo-HSCT. Patients 4, 7, and 9 maintained MRD negativity after the first transfusion of CAR-T cells and received a second infusion of CD19 CAR-T cells as consolidation therapy. However, they relapsed with CD19-positive leukemia at 740, 597, and 238 days respectively after the first infusion and received a third transfusion of CAR-T cells after relapse. Patient 4 and Patient 7 only achieved partial remission (which is being maintained at this time) and then received other chemotherapy according to the results of the high-throughput drug sensitivity analysis strategy. The leukemia cells of Patient 7 were highly sensitive to bortezomib. Thus, he received bortezomib (1.3 mg/m^2^, weekly) combined with methylprednisolone and achieved MRD-positive remission at one month. However, Patient 4 had no response to chemotherapy. Patient 9 failed to respond to CD19 CAR-T cells and ultimately died.

All five BCR-ABL-positive patients were directly bridged to allo-HSCT with a median time to transplantation of 51 days from CAR-T cell infusion. None of the patients received any treatment after transplantation, and none have experienced hematologic relapse since then. The follow-up period after transplantation of these five patients was 409 days (range: 172–945 days). Two patients died due to transplant-related toxic effects, including severe infection and GVHD.

### Adverse events

**Fig. 2:**
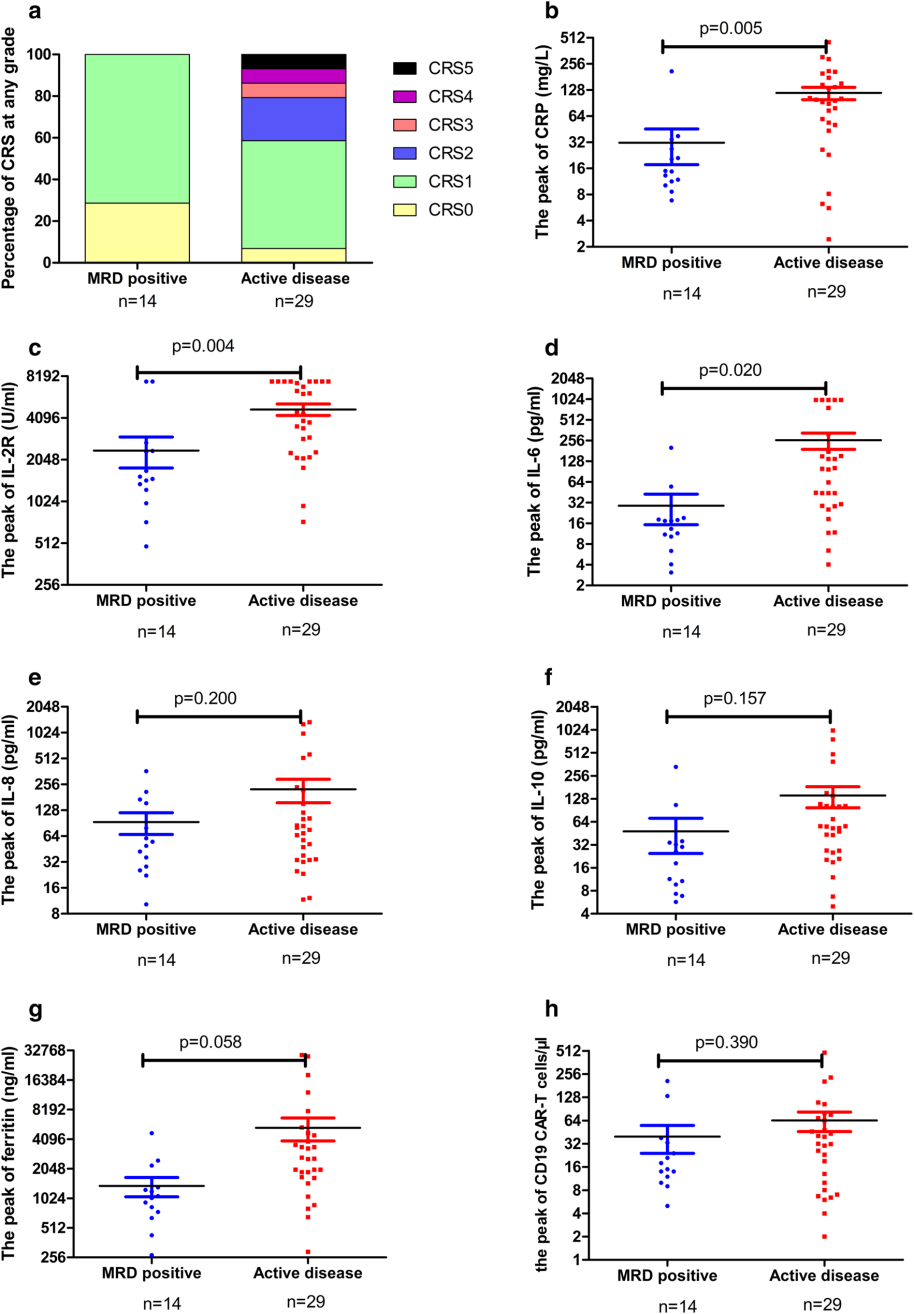
Comparison of cytokine release and CAR-T cell proliferation between MRD-positive patients and the patients with active disease after CAR-T cell infusion. **a** The incidence of CRS of all grades after CAR-T cell therapy in MRD-positive B-ALL patients (*n* = 14) and the patients with active disease (*n* = 29). **b**–**g** The peak levels of serum CRP, IL-2, IL-6, IL-8, IL-10 and ferritin in MRD-positive B-ALL patients (*n* = 14) or the patients with active disease (*n* = 29); **h** The peak level of CD19 CAR-T cells in peripheral blood after CAR-T cell infusion

The majority of adverse events after the first infusion were CRS and hematopoietic toxicities (Table 3). Most of the patients (71.4%) had grade 1 CRS after the first infusion. We also serially monitored serum cytokine levels in all patients. In contrast to the more robust cytokine response seen in relapsed/refractory B-ALL patients, in most MRD-positive patients, CAR-T yielded only a mild cytokine response [3, 13]. The degree of cytokine release was associated with fevers after CAR-T cell infusion (supplementary Fig. 2). Two patients with persistent high fever (Patients 5 and 7) had no response to antipyretics and were treated with steroids (supplementary Fig. 2a). Moreover, they showed a notable elevation in CRP, IL-2R, IL-6, IL-8, IL-10 and ferritin compared with other patients (supplementary Fig. 2b–g). Some studies have shown that corticosteroids might not influence the efficacy of CAR-T cells in B-ALL [14]. Two patients received steroid therapy to ameliorate CRS, and this treatment rapidly ameliorated their symptoms and cytokine levels. Patient 9 suffered a grade 2 CRS and robust release of cytokines after the third infusion of CAR-T cells after relapse, which may be due to the higher tumor burden in these patients. Compared to the patients with active disease, MRD-positive patients had a lower grade of CRS, and the peak levels of CRP, IL-2R and IL-6 were also significantly lower (Fig. 2a–g).

**Table 3:** Adverse events after first infusion of CAR-T cells

Adverse event	Grade 1, %	Grade 2, %	Grade 3,%	Grade 4, %
Inflammation-related event				
Fever	57.1	14.3		
Febrile neutropenia			57.1	
Cytokine release syndrome	71.4			
Hematological event				
Anemia	28.6	28.6	7.1	7.1
Leukopenia		42.9	42.9	14.3
Neutropenia	7.1	21.4	35.7	21.4
Lymphopenia		14.3	7.1	78.6
Thrombocytopenia	35.7	7.1	7.1	7.1
Fibrinogen decreased	7.1	7.1		
Nervous system event				
Chemical laboratory abnormalities				
Alanine aminotransferase increased	21.4			
Aspartate aminotransferase increased	7.1			
Gamma glutamyl transpeptidase increased	7.1			
Blood bilirubin increased	7.1			
Serum creatinine increased

### CAR-T cell expansion and peripheral blood lymphocyte subsets

Continuous monitoring of CAR-T cell expansion in peripheral blood suggested that the peak expansion of CAR-T cells occurred within 7–14 days and the number decreased to low or undetectable levels by 3 months (supplementary Fig. 2h). Consistent with the elevation of cytokines, the number of CAR-T cells was increased significantly in Patients 5 and 7. The peak expansion of CAR-T cells seemed to be lower in patients treated for MRD-positive disease in comparison to the active disease, but not that it is significant. (Fig. 2h).

B cell aplasia occurred in five patients who attained an MRD-negative CR after one cycle of CAR-T cell transfusion (Patients 3, 5, 7, 13 and 14; supplementary Fig. 3a, b). In contrast, two patients (Patients 11 and 12) who had no response to CAR-T therapy only exhibit a transient reduction in B cells. These results suggested that CAR-T cells could lead to persistent B cell depletion in MRD-positive B-ALL patients who responded. By comparing the frequencies and absolute numbers of T lymphocyte subsets between persistent CR patients and relapsed patients, we found that there was no significant difference in the frequencies or absolute numbers of T lymphocyte subsets (supplementary Fig. 3c, d).

### Effect of repeated infusion of CAR-T cells

**Fig. 3:**
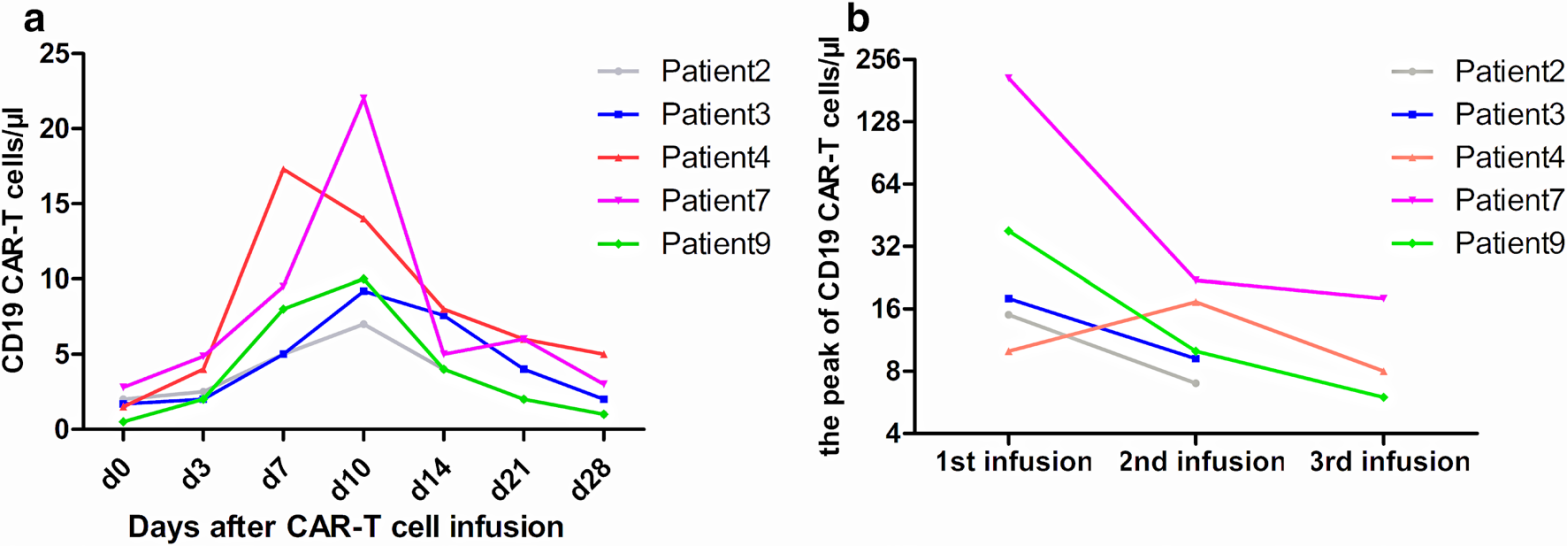
Proliferation of CAR-T cells after repeated infusion. **a** The proliferation and persistence of CAR-T cells after the second infusion; **b** The peak expansion of CAR-T cells after each infusion

In our study, five patients received two or more CAR-T cell infusions. One patient received a second CAR-T cell infusion in an MRD-negative status as consolidation therapy. The median infusion interval was 6.8 months (5.9–7.6 months). Except for Patient 9, who relapsed with CD19-positive leukemia at 53 days after the second infusion, the other four patients maintained a long period of MRD-negative state. However, three of the five patients ultimately had CD19- positive relapse and had no response to a third infusion. Moreover, repeated CAR-T cell infusion only resulted in mild proliferation of CAR-T cells (Fig. 3).

## Discussion

Although the CR rate of adult B-ALL is higher than 80%, a proportion of patients will eventually relapse due to residual leukemia cells in the bone marrow. These residual leukemia cells are called MRD. Several meta-analyses have shown that MRD alone is an indicator of poor prognosis and might be used to make individual treatment decisions for adult B-ALL patients [1]. Our study demonstrated that CAR-T cell infusion could rapidly eradiate leukemia cells and achieve sustained complete remission in patients with chemotherapy-refractory MRD. Moreover, CRS was less severe in patients with MRD than overt leukemia.

A number of studies have shown that CAR-T cells can rapidly induce molecular remission in relapsed or refractory B-ALL patients [15]. Although the clinical response to CAR-T cells in refractory or relapsed B-ALL is impressive, relapse after CAR-T cell treatment reduces their effectiveness. In patients with low tumor burden, CAR-T cells may have a good effect. Recently, Cheng et al. reported that donor-derived allogeneic CAR-T cells could be a useful way to eliminate MRD after allo-HSCT [9]. Moreover, Park et al. reported that the CRS rate in 21 ALL patients with MRD-negative or MRD positive CR were significantly lower than those in 32 patients with higher tumor burden. Our study showed that, after one cycle of CAR-T cell infusion, most of the patients with MRD positivity experienced rapid elimination of residual leukemia cells and achieved MRD-negative CR. Eight patients exhibited molecular resistance to previous chemotherapy, and CAR-T cells induced MRD-negative remission regardless of the extent of disease burden. By comparing the outcomes of patients with MRD positivity to those of the patients with active disease, we found that the probability of 2-year OS of B-ALL patients with MRD was higher than that of the patients with active disease, which indicated a better outcome in MRD patients treated with CAR-T cells. Moreover, the rate of CRS and cytokine release were mild in MRD-positive patients, which reduced CAR-T cell related mortality. These results support the notion that CAR-T cells may be a potentially curative treatment option for MRD-positive B-ALL patients.

It should be mentioned that most of the patients in our study received additional therapy (allo-HSCT or chemotherapy) after CAR-T infusion. Although the early response rate after CAR-T therapy represents the individual effect of CAR-T, the long-term favorable EFS and OS may represent the combination effect of CAR-T and additional therapy. In our study, four patients (Patient 4, 7, 8, 13) did not received additional therapy after CAR-T therapy, two of them (Patient 4,7) finally relapsed and received salvage chemotherapy. Other four patients who received only two or three cycle of chemotherapy before CAR-T treatment underwent consolidation and maintenance chemotherapy thereafter to prevent relapse. However, two of the four patients still relapsed. Thus, the limited data may indicate that patients may not benefit from additional chemotherapy after CAR-T therapy.

Although CAR T cells have good efficacy in B-ALL, the sustained remission after CAR-T is still an important issue and relapses remain a limitation of the therapy [7, 16]. Compared with chemotherapy and monoclonal antibodies, CD19 CAR-T cells showed a greater efficacy and long-lasting response [17, 18]. However, some patients still cannot achieve a sustained remission. The short persistence of CAR-T cells, CD19-negative escape and the inhibitory bone marrow environment may contribute to the relapse after CAR-T treatment. CAR-T with a 4-1BB costimulatory domain was proved to have better persistence than CAR-T with a CD28 costimulatory domain [19]. A recent study reported long-term outcomes from patients receiving CD19 CART with a 4-1BB costimulatory domain. The leukemia-free survival was 58% at 12 months [20]. Although the long-term benefit has been proved, strategies decreasing the relapse rate are still needed. Among the patients with high risk of relapse, allo-HSCT after CAR-T therapy may provide a promising strategy [21, 22]. MRD status at the time of allo-HSCT is strongly correlated with clinical outcome [23]. Eradication of residual leukemic cells before HSCT is beneficial for B-ALL patients. Unfortunately, some patients fail to achieve an MRD-negative CR before allo-HSCT due to chemotherapy resistance. In the present study, all five BCR-ABL-positive patients converted to MRD negativity after CAR-T cell transfusion and were offered subsequent allo-HSCT. Another BCR-ABL negative patient also underwent allo-HSCT. No clinical relapses have been observed to date. This result is consistent with previous studies, which showed that subsequent HSCT could improve the duration of remission after CAR-T cell therapy [8, 24, 25]. Thus, it seems that allogeneic HSCT following CAR-T treatment may be an effective way to eradicate residual leukemia cells and allogeneic HSCT instead of chemotherapy may be a good way to maintain long-term leukemia-free survival after CAR-T therapy. However, it should be noted that transplant-related mortality may offset the benefits of potential advantages.

Some studies have shown that patients with high MRD levels are more likely than patients with low MRD levels to develop hematologic relapse despite continued chemotherapy [1, 2, 26]. Of the eight patients who did not undergo subsequent transplantation for certain reasons, four patients suffered clinical relapse of medullary origin. One patient suffered CD19-negative relapse, and the other three suffered CD19-positive relapses. Pearson correlation analysis showed that the MRD level was negatively correlated with duration time of response. These data supported that higher MRD level may decrease the efficacy of CAR-T cells. With a median follow-up period of 647 days, the probability of EFS was 61.2% ± 14.0%. This result is better than that reported in previous studies about MRD-positive patients treated with chemotherapies or monoclonal antibodies [27–31]. Thus, patients with MRD-positive B-ALL can achieve long-term EFS after CAR-T cell treatment. CAR-T cells are a highly promising treatment that might be used as consolidation therapy to prevent relapse.

CRS is a major adverse effect that impedes the application of CAR-T cell therapy [32]. In our study, the severity of CRS was lower than that reported in relapsed/refractory B-ALL patients. This may be a result of the decreased tumor burden in MRD-positive patients who were treated with CAR-T cells. Some studies reported a high dose of steroid therapy for CRS might limit the proliferation of CAR-T cells and lead to immune escape. Another study showed that steroids did not influence the efficacy of CAR-T cells in B-ALL [14]. In our study, no patients in the MRD-positive group had grade 2 or higher CRS. Two patients were treated with steroid therapy to ameliorate cytokine-associated toxicities: one (Patient 5) relapsed with CD19-negative blasts, and the other relapsed with CD19-positive blasts. It seems that CD19 antigen loss might mainly be responsible for the relapse of the one patient. In summary, compared with relapsed/refractory patients, MRD-positive patients can receive not only favorable curative effects from CAR-T cell therapy but also decreased adverse effects.

Some studies have shown that repeated infusion of murine single-chain variable fragments can lead to immune rejection, which may be the cause of retreatment failure [33]. Our study showed that the three patients who received a third infusion of CAR-T cells after relapse showed no response or only partial response to the reinfusion. Meanwhile, the peak level of CAR-T cells after the third infusion was lower than that after the previous infusion. Immune rejection may contribute to the decreased expansion of CAR-T cells after reinfusion.

The main limitations of the study are the small number of patients and the short follow-up period. Another limitation is the retrospective design of the study. Despite these limitations, our work demonstrates that autologous CAR-T cells are effective in eradiating chemotherapy-resistant residual leukemia cells and are a superior treatment option for patients who are unable to receive intensive chemotherapy or transplantation. Furthermore, using CAR-T cells to bridge to allo-HSCT may dramatically improve the prognosis of these patients. The clinical benefits require further confirmation in a large randomized controlled trial and our center is initiating a prospective clinical trial to prove the beneficial effect of CAR-T cells in newly diagnosed high-risk B-ALL patients (ChiCTR1800015164).

In summary, CAR-T cell therapy seems to have a favorable curative effect but little toxicity in MRD-positive B-ALL patients. Moreover, patients can achieve sustained remission without transplantation. It may offer a novel and promising tool for relapsed/refractory MRD-positive B-ALL patients who are unable to undergo allo-HSCT. These preliminary results may serve as the basis for further prospective trials to demonstrate the benefits of CAR-T cell therapy. Whether CAR-T cell treatment will replace well-established HSCT or standard-of-care chemotherapy is still a question. A better understanding of the potential mechanisms of resistance to CAR-T cells, combination of CAR-T cells with other novel targeted drugs, and optimization of the manufacturing process will help to improve the outcomes of CAR-T cell therapy.

### Supplementary Information

Below is the link to the electronic supplementary material.

Supplementary file1 (PDF 722 kb)

## Abbreviations

allo-HSCT: Allogeneic hematopoietic stem cell transplantation
B-ALL: B-cell acute lymphoblastic leukemia
CD19 CAR-T: CD19-directed chimeric antigen receptor T
CR: Complete remission
CRP: C-reactive protein
CRS: Cytokine release syndromes
FCM: Flow cytometry
MRD: Minimal residual disease
NGS: Next-generation sequencing
OS: Overall survival
EFS: Event-free survival
RQ-PCR: Real-time quantitative polymerase chain reaction

## Declarations

### Availability of data and material

All data generated or analyzed during this study are included in this article.

## References

[1] Bassan R, Bruggemann M, Radcliffe HS, Hartfield E, Kreuzbauer G, Wetten S (2019). A systematic literature review and meta-analysis of minimal residual disease as a prognostic indicator in adult B-cell acute lymphoblastic leukemia. Haematologica.

[2] Raff T, Gokbuget N, Luschen S, Reutzel R, Ritgen M, Irmer S, Bottcher S, Horst HA, Kneba M, Hoelzer D, Bruggemann M, Group GS (2007). Molecular relapse in adult standard-risk ALL patients detected by prospective MRD monitoring during and after maintenance treatment: data from the GMALL 06/99 and 07/03 trials. Blood.

[3] Grupp SA, Kalos M, Barrett D, Aplenc R, Porter DL, Rheingold SR, Teachey DT, Chew A, Hauck B, Wright JF, Milone MC, Levine BL, June CH (2013). Chimeric antigen receptor-modified T cells for acute lymphoid leukemia. N Engl J Med.

[4] Davila ML, Brentjens RJ (2016). CD19-Targeted CAR T cells as novel cancer immunotherapy for relapsed or refractory B-cell acute lymphoblastic leukemia. Clin Adv Hematol Oncol.

[5] He X, Xiao X, Li Q, Jiang Y, Cao Y, Sun R, Jin X, Yuan T, Meng J, Ma L, Lu W, Lyu C, Liu K, Zhao M (2019). Anti-CD19 CAR-T as a feasible and safe treatment against central nervous system leukemia after intrathecal chemotherapy in adults with relapsed or refractory B-ALL [letter]. Leukemia.

[6] Maude SL, Laetsch TW, Buechner J, Rives S, Boyer M, Bittencourt H, Bader P, Verneris MR, Stefanski HE, Myers GD, Qayed M, De Moerloose B, Hiramatsu H, Schlis K, Davis KL, Martin PL, Nemecek ER, Yanik GA, Peters C, Baruchel A, Boissel N, Mechinaud F, Balduzzi A, Krueger J, June CH, Levine BL, Wood P, Taran T, Leung M, Mueller KT, Zhang Y, Sen K, Lebwohl D, Pulsipher MA, Grupp SA (2018). Tisagenlecleucel in children and young adults with B-cell lymphoblastic leukemia. N Engl J Med.

[7] Park JH, Riviere I, Gonen M, Wang X, Senechal B, Curran KJ, Sauter C, Wang Y, Santomasso B, Mead E, Roshal M, Maslak P, Davila M, Brentjens RJ, Sadelain M (2018). Long-term follow-up of CD19 CAR therapy in acute lymphoblastic leukemia. N Engl J Med.

[8] Lee DW, III, Stetler-Stevenson M, Yuan CM, Shah NN, Delbrook C, Yates B, Zhang H, Zhang L, Kochenderfer JN, Rosenberg SA, Fry TJ, Stroncek D, Mackall CL (2016) Long-term outcomes following CD19 CAR T cell therapy for B-ALL are superior in patients receiving a fludarabine/cyclophosphamide preparative regimen and post-car hematopoietic stem cell transplantation [abstract]. Blood 128 (22): Abstract 218

[9] Cheng Y, Chen Y, Yan C, Wang Y, Zhao X, Chen Y, Han W, Xu L, Zhang X, Liu K, Wang S, Chang L, Xiao L, Huang X (2019). Donor-derived CD19-targeted T cell infusion eliminates B cell acute lymphoblastic leukemia minimal residual disease with no response to donor lymphocytes after allogeneic hematopoietic stem cell transplantation. Engineering.

[10] Lee DW, Gardner R, Porter DL, Louis CU, Ahmed N, Jensen M, Grupp SA, Mackall CL (2014). Current concepts in the diagnosis and management of cytokine release syndrome. Blood.

[11] Neelapu SS, Tummala S, Kebriaei P, Wierda W, Gutierrez C, Locke FL, Komanduri KV, Lin Y, Jain N, Daver N, Westin J, Gulbis AM, Loghin ME, de Groot JF, Adkins S, Davis SE, Rezvani K, Hwu P, Shpall EJ (2018). Chimeric antigen receptor T-cell therapy—assessment and management of toxicities. Nat Rev Clin Oncol.

[12] Jin X, Cao Y, Wang L, Sun R, Cheng L, He X, Xiao X, Jiang Y, Li Q, Zhang H, Lu W, Lyu C, Jiang Y, Meng J, Zhao M (2020). HLA-matched and HLA-haploidentical allogeneic CD19-directed chimeric antigen receptor T-cell infusions are feasible in relapsed or refractory B-cell acute lymphoblastic leukemia before hematopoietic stem cell transplantation [letter]. Leukemia.

[13] Davila ML, Riviere I, Wang X, Bartido S, Park J, Curran K, Chung SS, Stefanski J, Borquez-Ojeda O, Olszewska M, Qu J, Wasielewska T, He Q, Fink M, Shinglot H, Youssif M, Satter M, Wang Y, Hosey J, Quintanilla H, Halton E, Bernal Y, Bouhassira DC, Arcila ME, Gonen M, Roboz GJ, Maslak P, Douer D, Frattini MG, Giralt S, Sadelain M, Brentjens R (2014). Efficacy and toxicity management of 19–28z CAR T cell therapy in B cell acute lymphoblastic leukemia. Sci Transl Med.

[14] Liu SY, Deng BP, PAN J, Yin ZC, Lin YH, Ling ZJ, Wu T, Gao ZY, Song YZ, Zhao YQ, Tong CR (2019) Corticosteroids do not influence the efficacy and kinetics of CAR-T cells for B-cell acute lymphoblastic leukemia [Abstract]. Blood 134 (Supplement_1): Abstract 228 10.1038/s41408-020-0280-y PMC7005173 32029707

[15] Brentjens RJ, Davila ML, Riviere I, Park J, Wang X, Cowell LG, Bartido S, Stefanski J, Taylor C, Olszewska M, Borquez-Ojeda O, Qu J, Wasielewska T, He Q, Bernal Y, Rijo IV, Hedvat C, Kobos R, Curran K, Steinherz P, Jurcic J, Rosenblat T, Maslak P, Frattini M, Sadelain M (2013). CD19-targeted T cells rapidly induce molecular remissions in adults with chemotherapy-refractory acute lymphoblastic leukemia. Sci Transl Med.

[16] Maude SL, Teachey DT, Porter DL, Grupp SA (2015). CD19-targeted chimeric antigen receptor T-cell therapy for acute lymphoblastic leukemia. Blood.

[17] Wei G, Hu Y, Pu C, Yu J, Luo Y, Shi J, Cui Q, Wu W, Wang J, Xiao L, Wu Z, Huang H (2018). CD19 targeted CAR-T therapy versus chemotherapy in re-induction treatment of refractory/relapsed acute lymphoblastic leukemia: results of a case-controlled study. Ann Hematol.

[18] Amrolia PJ, Pule M (2015). Chimeric antigen receptor T cells for ALL. Lancet.

[19] Zhao X, Yang J, Zhang X, Lu XA, Xiong M, Zhang J, Zhou X, Qi F, He T, Ding Y, Hu X, De Smet F, Lu P, Huang X (2020). Efficacy and safety of CD28- or 4–1BB-based CD19 CAR-T cells in B Cell acute lymphoblastic leukemia. Mol Ther Oncol.

[20] Zhang X, Lu XA, Yang J, Zhang G, Li J, Song L, Su Y, Shi Y, Zhang M, He J, Song D, Lv F, Li W, Wu Y, Wang H, Liu H, Zhou X, He T, Lu P (2020). Efficacy and safety of anti-CD19 CAR T-cell therapy in 110 patients with B-cell acute lymphoblastic leukemia with high-risk features. Blood Adv.

[21] Liu J, Zhang X, Zhong JF, Zhang C (2017). CAR-T cells and allogeneic hematopoietic stem cell transplantation for relapsed/refractory B-cell acute lymphoblastic leukemia. Immunotherapy.

[22] Hu L, Charwudzi A, Li Q, Zhu W, Tao Q, Xiong S, Zhai Z (2021). Anti-CD19 CAR-T cell therapy bridge to HSCT decreases the relapse rate and improves the long-term survival of R/R B-ALL patients: a systematic review and meta-analysis. Ann Hematol.

[23] Zhao XS, Liu YR, Xu LP, Wang Y, Zhang XH, Chen H, Chen YH, Han W, Sun YQ, Yan CH, Mo XD, Wang YZ, Fan QZ, Wang XY, Liu KY, Huang XJ, Chang YJ (2019). Minimal residual disease status determined by multiparametric flow cytometry pretransplantation predicts the outcome of patients with ALL receiving unmanipulated haploidentical allografts. Am J Hematol.

[24] Liu J, Zhang X, Zhong JF, Zhang C (2019). Use of chimeric antigen receptor T cells in allogeneic hematopoietic stem cell transplantation. Immunotherapy.

[25] Schubert ML, Huckelhoven A, Hoffmann JM, Schmitt A, Wuchter P, Sellner L, Hofmann S, Ho AD, Dreger P, Schmitt M (2016). Chimeric antigen receptor T cell therapy targeting CD19-positive leukemia and lymphoma in the context of stem cell transplantation. Hum Gene Ther.

[26] Bassan R, Spinelli O, Oldani E, Intermesoli T, Tosi M, Peruta B, Rossi G, Borlenghi E, Pogliani EM, Terruzzi E, Fabris P, Cassibba V, Lambertenghi-Deliliers G, Cortelezzi A, Bosi A, Gianfaldoni G, Ciceri F, Bernardi M, Gallamini A, Mattei D, Di Bona E, Romani C, Scattolin AM, Barbui T, Rambaldi A (2009). Improved risk classification for risk-specific therapy based on the molecular study of minimal residual disease (MRD) in adult acute lymphoblastic leukemia (ALL). Blood.

[27] Gokbuget N, Dombret H, Bonifacio M, Reichle A, Graux C, Faul C, Diedrich H, Topp MS, Bruggemann M, Horst HA, Havelange V, Stieglmaier J, Wessels H, Haddad V, Benjamin JE, Zugmaier G, Nagorsen D, Bargou RC (2018). Blinatumomab for minimal residual disease in adults with B-cell precursor acute lymphoblastic leukemia. Blood.

[28] Jen EY, Xu Q, Schetter A, Przepiorka D, Shen YL, Roscoe D, Sridhara R, Deisseroth A, Philip R, Farrell AT, Pazdur R (2019). FDA approval: blinatumomab for patients with B-cell precursor acute lymphoblastic leukemia in morphologic remission with minimal residual disease. Clin Cancer Res.

[29] Foa R, Vitale A, Vignetti M, Meloni G, Guarini A, De Propris MS, Elia L, Paoloni F, Fazi P, Cimino G, Nobile F, Ferrara F, Castagnola C, Sica S, Leoni P, Zuffa E, Fozza C, Luppi M, Candoni A, Iacobucci I, Soverini S, Mandelli F, Martinelli G, Baccarani M, Party GALW (2011). Dasatinib as first-line treatment for adult patients with Philadelphia chromosome-positive acute lymphoblastic leukemia. Blood.

[30] Kantarjian H, Stein A, Gokbuget N, Fielding AK, Schuh AC, Ribera JM, Wei A, Dombret H, Foa R, Bassan R, Arslan O, Sanz MA, Bergeron J, Demirkan F, Lech-Maranda E, Rambaldi A, Thomas X, Horst HA, Bruggemann M, Klapper W, Wood BL, Fleishman A, Nagorsen D, Holland C, Zimmerman Z, Topp MS (2017). Blinatumomab versus chemotherapy for advanced acute lymphoblastic leukemia. N Engl J Med.

[31] King AC, Pappacena JJ, Tallman MS, Park JH, Geyer MB (2019). Blinatumomab administered concurrently with oral tyrosine kinase inhibitor therapy is a well-tolerated consolidation strategy and eradicates measurable residual disease in adults with Philadelphia chromosome positive acute lymphoblastic leukemia. Leuk Res.

[32] Xiao X, He X, Li Q, Zhang H, Meng J, Jiang Y, Deng Q, Zhao M (2019). Plasma Exchange can be an alternative therapeutic modality for severe cytokine release syndrome after chimeric antigen receptor-T cell infusion: a case report. Clin Cancer Res.

[33] Turtle CJ, Hanafi LA, Berger C, Gooley TA, Cherian S, Hudecek M, Sommermeyer D, Melville K, Pender B, Budiarto TM, Robinson E, Steevens NN, Chaney C, Soma L, Chen X, Yeung C, Wood B, Li D, Cao J, Heimfeld S, Jensen MC, Riddell SR, Maloney DG (2016). CD19 CAR-T cells of defined CD4+:CD8+ composition in adult B cell ALL patients. J Clin Investig.

