# Supplementary material for: CD19 CAR-T cell treatment conferred sustained remission in B-ALL patients with minimal residual disease
Source: Cancer Immunol Immunother. 2021 Apr 25;70(12):3501–11. doi: 10.1007/s00262-021-02941-4 (PMC8571234; doi:10.1007/s00262-021-02941-4)
Supplement: Supplementary file 1 — Supplementary file1 (PDF 722 kb) [file 262_2021_2941_MOESM1_ESM.pdf]

supplementary Figure1

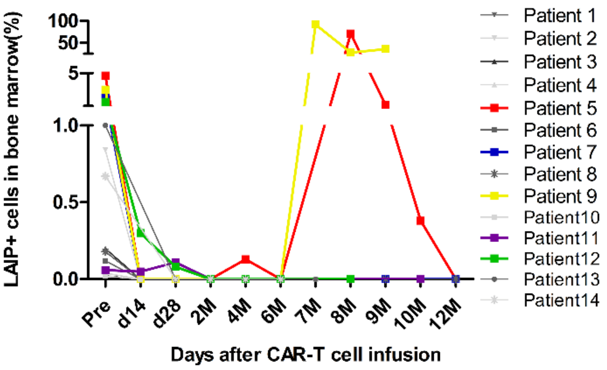

MRD level in the bone marrow of patients before and after CAR-T infusion.

LAIP: leukemia-associated antigen phenotype

supplementary Figure2

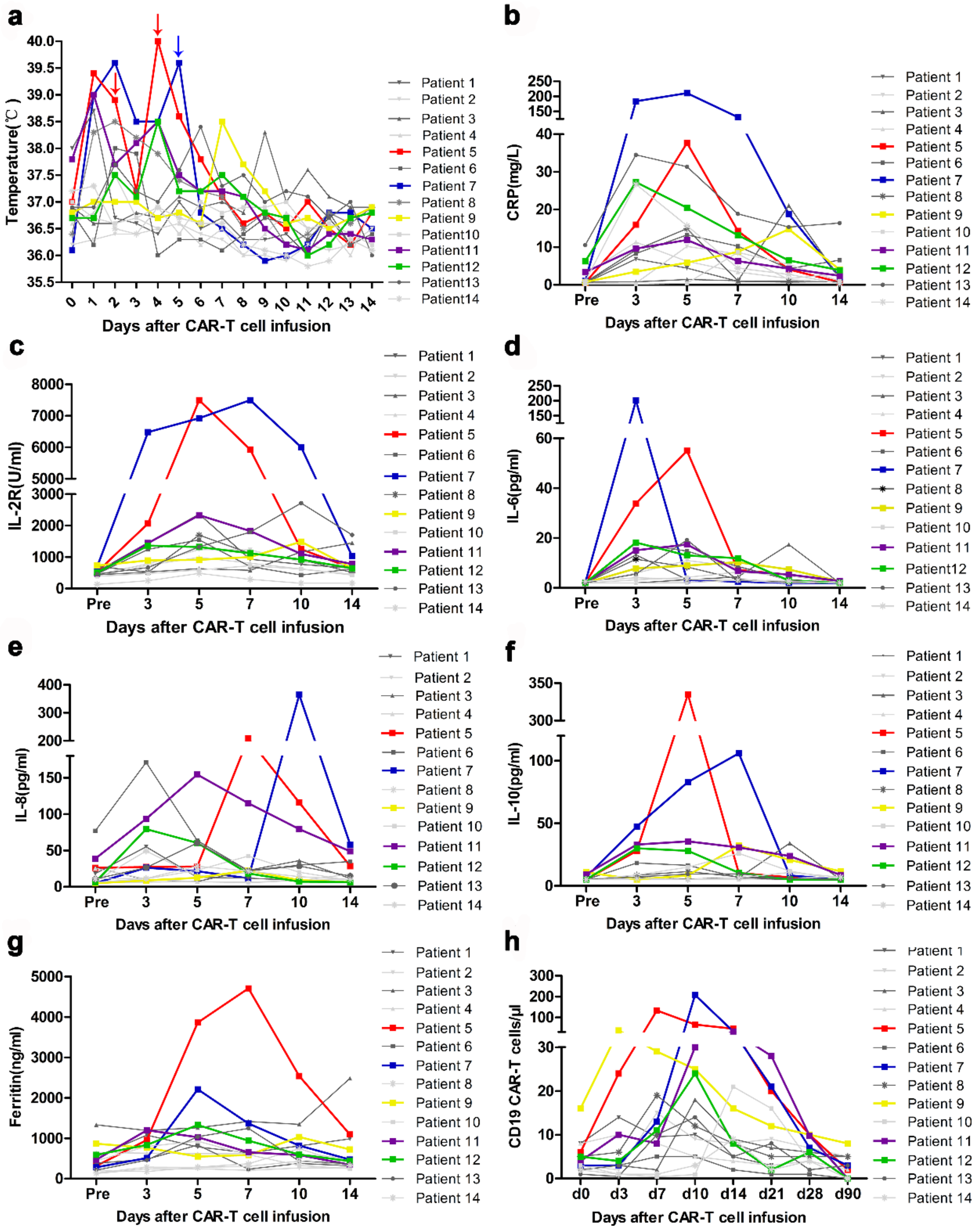

Cytokine release and CAR-T cell persistence in B-ALL patients after CD19 CAR-T cell therapy.

(a) Change in body temperature of B-ALL patients after infusion with CD19 CAR-T cells. The red arrows indicate steroid treatment in Patient 5. The blue arrow indicates steroid treatment in Patient 7; (b-g) The levels of serum CRP, IL-2, IL-6, IL-8, IL-10 and ferritin in B-ALL patients before and after CD19 CAR-T cell treatment; (h) The proliferation and persistence of CD19 CAR-T cells in peripheral blood after CAR-T infusion. The peak expansion of CAR-T cells occurred within 7 to 14 days, and the number of CAR-T cells decreased to low or undetectable levels by 3 months

supplementary Figure3

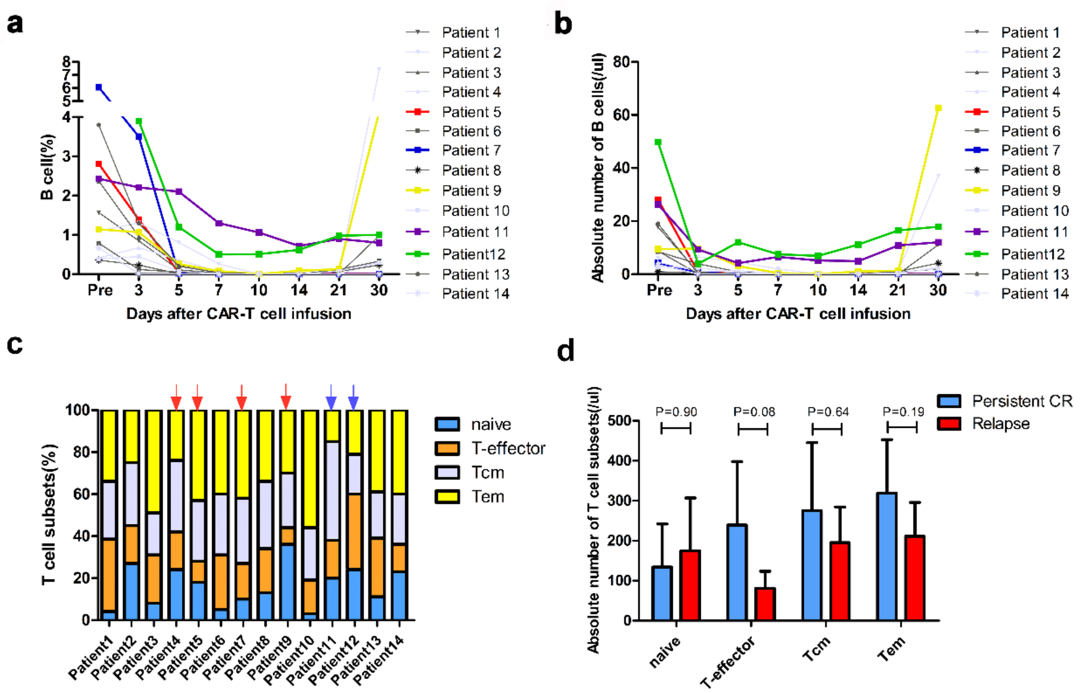

Classification of peripheral blood lymphocytes and T cells after CAR-T cell therapy.

The frequency (a) and absolute number (b) of B cells after CAR-T therapy; (c) The frequencies of T cell subsets at day 30 after CAR-T cell infusion. The red arrows indicate relapsed patients. The proportion of effector memory T cells (Tem) cells seemed lower in Patients 11 and 12 (blue arrows), who had no response to CAR-T cell infusion, than in other patients; (d) The absolute numbers of T cell subsets in patients with persistent CR or relapsed leukemia at day 30
